# Editorial: Quantitative MRI of blood-tissue interactions in the brain

**DOI:** 10.3389/fnins.2022.992427

**Published:** 2022-08-22

**Authors:** Jing Guo, Stefan Hetzer, Philipp Boehm-Sturm, Jens Wuerfel

**Affiliations:** ^1^Department of Radiology, Charité-Universitätsmedizin Berlin, Corporate Member of Freie Universität Berlin and Humboldt-Universität zu Berlin, Berlin, Germany; ^2^Berlin Center for Advanced Neuroimaging (BCAN), Charité-Universitätsmedizin Berlin, Corporate Member of Freie Universität Berlin and Humboldt-Universität zu Berlin, Berlin, Germany; ^3^Department of Experimental Neurology and Center for Stroke Research Berlin, Charité - Universitätsmedizin Berlin, Corporate Member of Freie Universität Berlin and Humboldt-Universität zu Berlin, Berlin, Germany; ^4^NeuroCure Cluster of Excellence and Charité Core Facility 7T Experimental MRIs, Charité-Universitätsmedizin Berlin, Corporate Member of Freie Universität Berlin and Humboldt-Universität zu Berlin, Berlin, Germany; ^5^MIAC AG, Basel and qbig, Department of Biomedical Engineering, University of Basel, Basel, Switzerland

**Keywords:** viscoelastic properties, vascular architecture and permeability, quantitative MRI (qMRI), perfusion and diffusion imaging, arterial pulsatility, cerebral blood flow (CBF)

Cerebral blood flow and global brain perfusion provide the brain parenchyma with oxygen and the nutrition required to maintain its normal function. Regulatory mechanisms ensure adequate and stable blood flow and perfusion. Changes in the blood volume and the underlying vascular structure in turn alter the intracranial tissue mechanical properties such as stiffness and fluidity. Therefore, blood perfusion, water diffusion, intracranial pressure, and cerebral viscoelastic properties change synergistically during physiological and/or pathological blood flow responses. Investigation of these flow-related properties is of utmost importance to help us understand brain function in health and disease.

Novel quantitative magnetic resonance imaging (MRI) techniques for cerebral flow assessment have emerged, allowing us to probe the interactions between blood and brain tissue. Biophysical parameters such as cerebral biomechanical properties, microstructure, the blood volume fraction, blood flow, and the metabolic rate of oxygen can be obtained by MRI techniques such as magnetic resonance elastography (MRE), diffusion-weighted imaging (DWI), arterial spin labeling (ASL) perfusion imaging, and functional MRI (fMRI). Along with recent advancements in MRI data acquisition and reconstruction methods, these multiparametric imaging approaches provide tools for the sensitive characterization of cerebral blood-tissue interactions with high spatiotemporal resolution.

This editorial summarizes a set of articles dealing with “*Quantitative MRI of Blood-Tissue Interactions in the Brain*” that appeared in the Brain Imaging Methods section of *Frontiers in Neuroscience*. The researchers who contributed to this Research Topic presented eight original research articles highlighting the most recent advances in our understanding of the dynamic interplay between fluid and solid components in the brain. The biophysical properties of the brain and cerebral hemodynamics were investigated using numerical simulation as well as *in vivo* examinations in both human brains and animal models.

Characteristics of the blood brain barrier (BBB), such as its integrity and permeability, were examined by Mahroo et al. and Shao et al., respectively. Investigating BBB integrity, Mahroo et al. acquired *in vivo* data using a multiple echo time arterial spin labeling (multi-TE ASL) sequence and fitted the results with an extended model accounting for intravoxel transit time. These *in vivo* results, along with simulated data, demonstrated that the acquisition method and the extended model delivered good reproducibility and shorter exchange time (Texch), a surrogate for BBB integrity, compared with the two-compartment model. Shao et al. investigated BBB permeability by quantifying the water exchange rate (kw) using diffusion-prepared pseudo-continuous arterial spin labeling (DP-pCASL) and compared the results with those obtained by measuring the volume transfer constant (Ktrans) and the exchange rate of a gadolinium-based contrast agent (GBCA), commonly assessed by dynamic contrast-enhanced (DCE) MRI. As shown by *in vivo* data acquired in 16 elderly subjects, kw can be measured with good reproducibility. Interestingly, Shao et al. found significant correlations between kw and GBCA-based parameters in only three brain regions, assuming attributing their observations to the different transport mechanisms between water and GBCAs as well as the possible effects of differences in vascular structure between brain regions.

Changes in cerebral biophysical properties as a result of BBB disruption were explored from a biomechanical perspective by Silva et al. and Schregel et al. In both studies, mouse models of neuroinflammation were investigated, and *in vivo* cerebral viscoelasticity was quantified by MRE. In the study presented by Silva et al., GBCA and superparamagnetic iron oxide particles (VSOP) were used to label regions with BBB disruption and strong immune cell infiltration, respectively. Cerebral stiffness in these regions showed significant differences in the degree of softening comparing to control baseline values, making MRE a promising tool for detecting different pathological aspects of neuroinflammation. Schregel et al. developed a model of controlled focal inflammatory brain lesions using focused ultrasound (FUS). Induced by FUS, foci of activated microglia/macrophages were consistently observed in the sonicated hemisphere, which displayed lower stiffness compared to the normal hemisphere.

Whittaker, Steventon et al. contributed two articles assessing arterial pulsatility and dynamic cerebral autoregulation (dCA). In their first study, the authors proposed a new MRI method, dynamic inflow magnitude contrast (DIMAC), which is solely based on the inflow effect, to measure the real-time pulsatile flow in cerebral arteries. Changes in DIMAC signal contrast observed in hypercapnia and in response to the thigh-cuff release (TCR) challenge demonstrated the sensitivity of DIMAC to transient pulsatility variation as well as the steady-state in arterial tone. In the second study, Whittaker, Fasano et al. employed blood oxygen level-dependent (BOLD) fMRI to capture the flow response to the TCR challenge and mapped dCA using voxel-wise assessment of the hemodynamic response function (HRFTCR).

In the study presented by Malm et al., blood flow was quantified in patients with ischemic stroke using 4D flow MRI. In additional to assessing the feasibility of the method, the authors also reported the potential added value of 4D flow MRI compared with computed tomography angiography. Finally, using finite-element tissue mechanical modeling and simulated MRI data, Zoraghi et al. predicted small tissue deformations resulting from local blood flow changes and associated changes in brain stiffness and volume in activated cortical tissue. The authors also addressed the implications of the aforementioned tissue shifts on various fMRI methods and the necessity to characterize and to correct the resulting spurious fMRI signal changes.

To conclude, the studies featured in this Research Topic gave an overview of our current understanding of the brain parenchymal biophysical properties and cerebral hemodynamics as quantified by multiparametric MRI. The authors highlighted the complex yet intriguing interaction between blood, the vasculature and brain tissue, deriving new insights into the role of blood flow and perfusion in maintaining brain function and health.

## Author contributions

JG wrote the editorial. SH, PB-S, and JW edited the editorial. All authors contributed to the article and approved the submitted version.

## Conflict of interest

The authors declare that the research was conducted in the absence of any commercial or financial relationships that could be construed as a potential conflict of interest.

## Publisher's note

All claims expressed in this article are solely those of the authors and do not necessarily represent those of their affiliated organizations, or those of the publisher, the editors and the reviewers. Any product that may be evaluated in this article, or claim that may be made by its manufacturer, is not guaranteed or endorsed by the publisher.

